# Reasoning on conflicting information: An empirical study of Formal Argumentation

**DOI:** 10.1371/journal.pone.0273225

**Published:** 2022-08-19

**Authors:** Mathieu Guillaume, Marcos Cramer, Leendert van der Torre, Christine Schiltz

**Affiliations:** 1 Cognitive Science and Assessment (COSA) Institute, University of Luxembourg, Esch-sur-Alzette, Luxembourg; 2 Graduate School of Education, Stanford University, Stanford, California, United States of America; 3 Computer Science and Communications (CSC), University of Luxembourg, Esch-sur-Alzette, Luxembourg; 4 International Center for Computational Logic, TU Dresden, Dresden, Germany; Bogazici University, TURKEY

## Abstract

According to *the Argumentative Theory*, human reasoning has an argumentative function, which consists of devising and evaluating arguments for and against various claims. It is however unclear how humans handle conflicting claims they face in everyday life (i.e., “Bob is telling me that Alice is at the library” vs. “Charles is telling me that Alice is at home”). We here investigate human argumentative reasoning in the light of *Formal Argumentation*, a research field that develops formal methods to give a normative account of argumentation and reasoning about conflicting information. In Formal Argumentation, multiple argumentation semantics that allow selecting sets of jointly acceptable arguments have been proposed. Nonetheless, it is unclear which of these semantics predicts best how humans evaluate the acceptability of conflicting arguments. We conducted an empirical study in which 130 young adults judged natural language arguments. We instructed them to draw the attack relation between the given arguments and to evaluate the acceptability of each of these arguments. Our results show that human judgments on the existence and directionality of attacks between the arguments conform to theoretical predictions from Formal Argumentation. We further found out that some less well-known argumentation semantics predicted human evaluation better than the most well-known semantics. These findings support the cognitive plausibility of variants of Formal Argumentation and bring new insights into reasoning about conflicting information.

## Introduction

Human reasoning is an important form of social and cognitive interaction [1]. It had long been studied through the lens of classic monotonic logic, irrespective of these interactions. For instance, Wason found out that adult participants seldom adopt the right behavior to verify whether an abstract rule in the form of “*if p then q*” is true, supporting that humans make many mistakes when reasoning about abstract material [2]. In this respect, extensive empirical evidence led researchers to agree that human reasoning is *irrational* (see [3] for a review). However, such a statement seems overstated from an evolutionary perspective (see [4] for a review), and one could alternatively conclude that classic monotonic logic fails to describe human reasoning. Other explanatory frameworks of human thinking and reasoning were thus explored. Some researchers proposed that human reasoning consists of constructing mental models to envisage the different possibilities and draw conclusions from them [5, 6]. In their *Argumentative Theory*, Mercier and Sperber put forward the argumentative function of reasoning [7]. According to these authors, reasoning is arguing, that is, devising and evaluating arguments to persuade. This theory emphasizes the need for explaining how humans argue and reason about conflicting information to understand human cognition. In the current study, we aimed at assessing whether *Formal Argumentation* theory [8] would provide new insights about how humans handle conflicting claims they are likely to face in everyday life (i.e., “Bob is telling me that Alice is at the library” vs. “Charles is telling me that Alice is at home”).

Over the past 35 years, there has been a surge in research within the field of *Artificial Intelligence* (AI) aimed at overcoming the limitations of classical logic, giving rise to the research fields of *Formal Argumentation*, introduced by Dung [8]. It is worth noting that Formal Argumentation consists of two major branches: In *Abstract Argumentation*, one models arguments by abstracting away from their internal structure to focus on determining acceptable arguments based on the attack relation between them. In *Structured Argumentation*, one additionally models the internal structure of arguments through a formal language in which arguments and counterarguments can be constructed (see [9], for an overview of different formalisms for Structured Argumentation).

Formal Argumentation develops formal methods based on logic and graph theory to provide a normative account of argumentation and reasoning about conflicting information [10]. It complements classical logic in the sense that classical logic by itself cannot properly grasp reasoning about conflicting information since it fails to capture why an argument that may be considered a good argument by itself may still become the target of a successful counterargument. Formal Argumentation can account for this feature, which makes this approach appropriate to model human reasoning, yet the theory has received little to no interest from cognitive scientists. This disinterest is somewhat unfortunate because Formal Argumentation provides an objective framework to analyze conflicting arguments and could grant critical insights about how humans deal with incompatible information.

### Attack relation in Formal Argumentation

One main point of focus in Formal Argumentation is the *attack relation* between arguments. An argument *A* is said to *attack* an argument *B* if *A* can be used as a counterargument against *B*. A critical observation due to Dung [8] is that under certain conditions, one can make rational judgments about the acceptability of arguments based only on the attack relation between them. As an example, consider the three following arguments:

*A*. *Yesterday*, *Alice said she would be at the library today*, *so we can find her in the library now*.
*B*. *John said that Alice fell ill this morning*, *so she cannot be at the library now*.
*C*. *John is a notorious liar who often makes up stories about his mother Alice*.

In this example, argument *B* attacks argument *A* because argument *B* can be used as a counterargument against argument *A*. Similarly, argument *C* attacks argument *B*. Argument *C* is not attacked by any argument and should thus be accepted. Since argument *C* attacks argument *B*, this leads to the rejection of argument *B*. Once argument *B* is rejected, there is no longer any acceptable argument that attacks argument *A*, and argument *A* can be accepted.

While classical logic presents us with a unified formal system, Formal Argumentation is characterized by the comparative study of competing formalisms and competing semantics for evaluating the acceptability of arguments. Therefore, unlike classical logic, Formal Argumentation generally does not provide a single norm of “correct” reasoning, but presents us with multiple competing norms, in the form of competing *argumentation semantics*. In this study, we investigated the cognitive plausibility of some key aspects of Formal Argumentation: On the one hand, we compared human judgments about the existence and directionality of attacks between arguments with theoretical predictions based on the ASPIC+ framework for Structured Argumentation [11]. On the other hand, we considered various argumentation semantics as predictors of human reasoning and compared their capacity to correctly predict human thinking. In the following section, we illustrate what is an argumentation framework and we explain the three argumentation semantics that we extensively refer to in the current study. We give detailed formal definitions and descriptions of the three semantics in S1 File.

### Argumentation frameworks and argumentation semantics

#### Illustration of Argumentation frameworks

**Fig 1:**
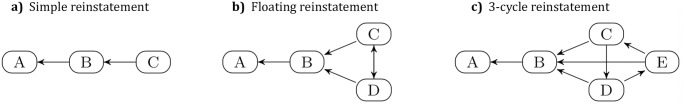
Illustration of the argumentation frameworks we considered in this study. The figure depicts a) Simple Reinstatement (*AFSimple*, left), b) Floating Reinstatement (*AFFloating*, center), and c) 3-cycle Reinstatement (*AF3-cycle*, *right*).

Dung introduced *Abstract Argumentation* as a formal method for studying the acceptability of arguments based on the attack relation between arguments [8]. Abstract argumentation is an abstraction and a simplification of regular argumentation, in which the internal structure of arguments is abstracted away to focus on the attack relation between the arguments and on determining the acceptability of arguments based only on this attack relation. A set of arguments together with an attack relation between them is called an *Argumentation Framework (AF)*. It is generally assumed that an argumentation framework contains all arguments and attacks that are relevant to the acceptability of the arguments in the framework. An *AF* can be graphically visualized by drawing an arrow from *A* to *B* to represent the fact that argument *A* attacks argument *B*. Fig 1 depicts the three *AF*s we used in this study.

The first Argumentation Framework depicted in Fig 1a expresses that there are three arguments, *A*, *B*, and *C*, and that argument *C* attacks argument *B*, which in turn attacks argument *A*. This AF corresponds to the example that we provided in the previous section. This framework is called *Simple Reinstatement* (*AF*_*Simple*_). The second AF in Fig 1b expresses that there are four arguments and that argument *D* attacks arguments *B* and *C*, argument *C* attacks arguments *B* and *D*, and argument *B* attacks argument *A*. To illustrate this second AF, let’s add the following argument D to our previous example:

*D*. *John is a notorious liar who often makes up stories about his daughter Alice*.

In this new set of four arguments, arguments C and D both attack argument B, but they also attack each other because Alice cannot simultaneously be John’s mother and daughter. This situation is called the *Floating Reinstatement* (*AF*_*Floating*_). In the third AF we used in this study, depicted in Fig 1c, there are three arguments, *C*, *D*, and *E* that attack argument *B*, which in turn attacks argument *A*; but also, *C* attacks *D*, *D* attacks *E*, and *E* attacks *C*. Such a triangular attack relation between *C*, *D*, and *E* is called a *3-cycle* (*AF*_*3-cycle*_). We provide an illustration of an *AF*_*3-cycle*_ in our S2 File.

Given an *AF*, one goal of Formal Argumentation is to select a set of arguments deemed acceptable on the sole basis of the attack relation between the arguments. One desideratum for such a set of arguments is that it is *conflict-free*, meaning that it does not contain two arguments amongst which one attacks the other. For example, for *AF*_*Simple*_, the set {*A*,*C*} is conflict-free, as *A* does not attack *C*, and *C* does not attack *A*. Abstract Argumentation provides multiple methods for choosing such sets of acceptable arguments that satisfy certain additional desiderata beyond conflict-freeness. An example of such an additional desideratum is that an argument that is not attacked by any argument should always be accepted. In *AF*_*Simple*_, this means that *C* should be accepted because it is not attacked by any argument.

A systematic method for choosing sets of jointly acceptable arguments from any given argumentation framework is called *argumentation semantics*. A set of arguments that are jointly acceptable according to a given semantics is called an *extension* of the argumentation framework in the given semantics. Baroni, Caminada, and Giacomin give an overview of the current state of the art of argumentation semantics [12]. In this study, we focused on three standard argumentation semantics from the abstract argumentation literature, namely *grounded semantics*, *preferred semantics*, and *CF2 semantics*, more precisely on the justification statuses of arguments with respect to these semantics. Note that we will not focus on two additional semantics widely considered in the literature: *complete* semantics and *stable* semantics (see [10, 13], for overviews). The justification status in *complete* semantics is the same as in *grounded* semantics, so we do not consider *complete* semantics separately. S*table* semantics has the serious disadvantage that for some *AF*s, there is no *stable* extension so that the justification statuses are undefined. We will now briefly provide an informal explanation of *grounded semantics*, *preferred semantics*, and *CF2 semantics* as well as the notion of *justification status*.

#### Argumentation semantics

*Grounded and preferred semantics*. In the case of AF_Simple_ depicted in Fig 1a, one can reason as follows: Argument C should be accepted because it is not attacked by any other argument. Once argument C is accepted, we can defend argument A against the attack from argument B because the already accepted argument C attacks argument B. In this case, we say that argument C defends argument A. Since argument C is already accepted and defends argument A, we also accept argument A. Argument B, on the other hand, should be rejected because it is attacked by the accepted argument C. Both *grounded* and *preferred* semantics are based on this idea of *defense* (see S1 File for more information).

Nevertheless, there are crucial differences between these two semantics: *Grounded* semantics formalizes a very skeptical approach to argument acceptance, whereby we only accept arguments when the kind of reasoning presented above forces us to accept the argument. In the case of the Floating Reinstatement and the 3-Cycle AF depicted in Fig 1b and 1c, no argument is not attacked, so there is no argument with which we can start the process of accepting arguments. Therefore, we do not accept any arguments. But since we have no way to justify the rejection of an argument through an accepted counterargument, we also do not reject any argument. Instead, we say that all arguments in AF_Floating_ and AF_3-cycle_ are *undecided* in *grounded* semantics. *Preferred* semantics is more lenient, in that it allows for jumping to conclusions about which arguments should be accepted. For example, in the case of the AF_Floating_, *preferred* semantics allows one to choose to accept argument C because once one accepts this argument, it can defend itself against the attack from argument D. But one can also make the choice to accept argument D, because once one accepts argument D, it can defend itself against the attack from argument C. Note that whichever of these two arguments we accept, we can reject argument B and thus defend argument A, which we, therefore, accept in either case. Overall, we have either the choice to accept arguments C and A or to accept arguments D and A.

When we have multiple choices in this way, we need a way to make a single judgment about each argument based on all possibilities. This single judgment about each argument is called the *justification status* of that argument. The idea here is that when an argument is accepted according to all possible choices, that justification status of the argument is *strongly accepted*. When an argument is attacked by an accepted argument according to all possible choices, the justification status of the argument is *strongly rejected*. When neither of these two conditions is satisfied, we say that the justification status of the argument in question is *undecided*. In the case of the AF_Floating_, this means that in *preferred* semantics, argument A is strongly accepted, argument B is strongly rejected, and arguments C and D are undecided.

Regarding the 3-cycle Reinstatement AF depicted in Fig 1c, one can reason as follows: If we try to accept argument C, then this leads to the rejection of argument D, which in turn means that argument E is defended and should therefore be accepted. But in this case, we would accept both argument C and argument E, even though argument E attacks argument C. Such an acceptance of two arguments that conflict with each other is not allowed. Therefore, we cannot accept argument C. By the same reasoning, arguments D and E can also not be accepted. However, we also cannot reject any of these three arguments, because the rejection of an argument must be justified through the acceptance of a counterargument. Accepting argument B would require being able to defend it from the attacks from C, D, and E, but without accepting any of these arguments, we cannot defend argument B, so argument B cannot be accepted. But argument B can also not be rejected, because no counterargument against it is accepted. By similar reasoning, argument A can also neither be accepted nor rejected. In the end, all five arguments are *undecided* in the *preferred* semantics.

*CF2 semantics*. While grounded *semantics* led to undecidedness about all arguments in the case of the AFs depicted in Fig 1b and 1c, *preferred* semantics allowed us to make decisions about arguments A and B in Fig 1b, but still led to undecidedness for all arguments in Fig 1c. The idea behind the CF2 semantics is to minimize the amount of undecidedness even further. For achieving this, one needs to drop the criterion that the accepted arguments must defend themselves against all attacks from other arguments. To get a meaningful decision about argument acceptability in the absence of this criterion, one strictly follows the directionality of the attack relation when accepting arguments according to the CF2 semantics. For example, in the case of *AF*_*3-cycle*_ depicted in Fig 1c, this means that we first make a decision about the arguments *C*, *D*, and *E* before making a decision about argument *B*, because we can move from arguments *C*, *D* and *E* towards argument *B* along with attack arrows that point in the direction of the movement, but we cannot move in this way from argument *B* to any of *C*, *D*, and *E*. For the same reason, we make a decision about argument *B* before making a decision about argument *A*. In the case of arguments *C*, *D*, and *E*, on the other hand, we need to make the decision about all three of them at the same time, because each of them can be reached in this way from any other one of them, so there is no way to say that one of them comes before the others.

Now the key idea behind the CF2 semantics is that when we are in this way forced to make a decision about multiple arguments at once, we choose to accept some of these arguments that are not in conflict with each other and to which no further of these arguments could be added without generating a conflict. Once such a choice has been made, we delete all arguments that are attacked by a chosen argument.

In the case of the AF depicted in Fig 1c, we first need to make a decision about arguments *C*, *D*, and *E*. Accepting any two of these arguments would lead to a conflict, so we accept only one of them. So here we have three possible choices, either accepting *C*, or accepting *D*, or accepting *E*. In each of these cases, argument *B* will be deleted, because it is attacked by an accepted argument. After the deletion of argument *B*, argument *A* is no longer attacked by any argument and is therefore accepted.

We now define the justification status in the same way as in *preferred* semantics. This means that in this example, argument *A* is strongly accepted, argument *B* is strongly rejected, and arguments *C*, *D*, and *E* are all undecided.

One can easily see that the same procedure applied to the AFs in Fig 1a and 1b leads to the same results for those AFs as in the case of the preferred semantics. Table 1 summarizes the results for all three considered AFs for the CF2 semantics. Note that all semantics agree on *AFSimple*.

**Table 1:** Summary of the extensions and justification status for each of the three semantics and the three *AF*s considered in this study.

		*grounded* semantics	*preferred* semantics	*CF2* semantics
***AF***_***Simple***_ (Fig 1A)				
Extensions		{A,C}	{A,C}	{A,C}
Justification status	Argument A	Strongly Accepted	Strongly Accepted	Strongly Accepted
	Argument B	Strongly Rejected	Strongly Rejected	Strongly Rejected
	Argument C	Strongly Accepted	Strongly Accepted	Strongly Accepted
***AF***_***Floating***_ (Fig 1B)				
Extensions		{}	{A,C} {A,D}	{A,C} {A,D}
Justification status	Argument A	Undecided	Strongly Accepted	Strongly Accepted
	Argument B	Undecided	Strongly Rejected	Strongly Rejected
	Argument C	Undecided	Undecided	Undecided
	Argument D	Undecided	Undecided	Undecided
***AF***_***3-cycle***_ (Fig 1C)				
Extensions		{}	{}	{A,C} {A,D} {A,E}
Justification status	Argument A	Undecided	Undecided	Strongly Accepted
	Argument B	Undecided	Undecided	Strongly Rejected
	Argument C	Undecided	Undecided	Undecided
	Argument D	Undecided	Undecided	Undecided
	Argument E	Undecided	Undecided	Undecided

### Related work

While Formal Argumentation is an important branch of research within AI, only a few studies have empirically investigated the cognitive plausibility of the formalisms from argumentation theory. The first of its kind was the study of Rahwan, Madakkatel, Bonnefon, Awan, and Abdallah [14], who tested how humans evaluate simple reinstatement and floating reinstatement. They found that confidence in a given argument A decreased when it was attacked by an argument B, but bounced back up when B was attacked by a third argument C. Findings were similar in the case of floating reinstatement, where confidence in an argument A decreased when it was attacked by an argument B, but also bounced back up when B was attacked by two competing arguments C and D. Being the first study to investigate the cognitive plausibility of the formalisms from argumentation theory, it laid the foundations for further work in this area. For instance, Cerutti, Tintarev, and Oren [15] tested the correspondence between the human evaluation of arguments and properties of a logic-programming-based approach to Structured Argumentation proposed by Prakken and Sartor [16]. Rosenfeld and Kraus [17] have empirically studied human argumentative behavior and compared it to bipolar argumentation frameworks [18]. Polberg and Hunter [19] performed an experiment to investigate the relationship between human reasoning on the one hand and bipolar and probabilistic approaches to abstract argumentation on the other hand. Of these four empirical studies, the aims of the study by Rahwan *et al*. [14] come closest to ours. Their paper includes a discussion of why this kind of empirical validation of formalisms from argumentation theory is a highly relevant method that complements the more widely applied example-based and principle-based approaches.

Nevertheless, the study of Rahwan *et al*. [14] had some limitations. For instance, in their study, participants were asked to assess the conclusion of a designated argument on a 7-point Likert scale from *certainly false* to *certainly true*. However, it is difficult to compare their 7-point Likert scale results to the predictions of argumentation formalisms that are two- or three-valued. Furthermore, the study from Rahwan et al. [14] only made use of two argumentation frameworks, namely the simple reinstatement framework and the floating reinstatement framework (see Fig 1). Since all standard semantics agree on the evaluation of simple reinstatement, only the results on floating reinstatement could distinguish between different semantics, so only limited claims could be made about which semantics best predicts human judgments. Finally, the authors did not empirically test their assumption that their natural language argument sets correspond to the intended argumentation frameworks. This limitation is especially pressing because the attacks that they intended to be unidirectional were based on conflicts between the conclusion of the attacking argument and the premise of the attacked argument, without any indication of preference. In the frameworks of Structured Argumentation from the ASPIC family [11, 20, 21], such underminings without preferences always give rise to bidirectional attacks. Empirical findings indeed confirmed that humans more frequently judged underminings as bidirectional rather than unilateral attacks [22].

### Objectives and hypotheses

In the current study, we intended to test the cognitive plausibility of Formal Argumentation. To do so, we conducted an empirical study in which young adults were instructed to judge conflicting natural language arguments.

We hypothesize that Formal Argumentation can predict human understanding and evaluation of arguments. We tested this hypothesis on two different tasks. First, we instructed participants to explicitly draw the attack relations between all arguments from a given set. The drawings allow verifying whether participants understood the conflicts between the arguments in accordance with theoretical predictions based on Structured Argumentation. We expected that the graphical responses of the participants would be–or become through group discussion–in line with these argumentation-theoretic predictions. In a second task, participants had to evaluate the acceptability status of each argument from the same set. In the sets where the three standard semantics agree (i.e., the natural language argument sets corresponding to *AFSimple*), we expected human participants to judge the acceptability status of conflicting arguments according to the predictions provided by Abstract Argumentation. Such observations would support our first hypothesis that Formal Argumentation is cognitively plausible. If this is the case, then we can extend the scope of our study by directly comparing the predictive capacity of the considered semantics. Subsequently, in the sets where the three semantics disagree (i.e., *AFFloating* and *AF3-cycle*), we can additionally investigate whether one of the three argumentation semantics defined above (*grounded*, *preferred*, and *CF2*) better predicts how humans judge the acceptability status of conflicting arguments. We aimed here at identifying which of the three semantics has the best predictive capacity, and consequently which variant is the most likely to be cognitively plausible.

Noteworthily, when faced with demanding reasoning tasks, humans often show a tendency to reduce the cognitive costs, for example by reinterpreting the task in a simplified way or by choosing the first solution that comes to their minds without checking it for internal consistency and adequacy as a solution to the presented task [23, 24]. We aimed at reducing this potential bias by studying how humans solve reasoning tasks when collaboratively thinking in a group, as such group deliberation is known to increase performance in logical reasoning tasks [25, 26]. Previous results showed that individual performance, which has generally been reported to be quite poor in pure logic and reasoning tasks, could be enhanced by a cooperative discussion with peers. For instance, faced with the Wason selection task [2], humans solving the task in groups achieved a level of insight that was qualitatively superior to the one achieved by single individuals [25, 26]. Additionally, and more generally, discussion with peers was proven to substantially improve motivation to solve a given task [27]. For these reasons, we decided to incorporate a cooperative discussion in our method to help participants to elaborate and enrich their thinking. This collective step was designed to obtain a more reliable evaluation of the justification status. Such reliability is crucial to test the cognitive plausibility of our predictions.

## Method

The Ethics Review Panel from the University of Luxembourg approved the method and the implementation of the experiment (comprising the pilot studies) before the start of data collection. Written consent from the participants was obtained.

### Participants

We recruited 130 undergraduate students (82 females, 48 males) from the University of Luxembourg through physical and online posters. Participants were aged from 18 to 36 years old (mean age = 22.01) and were from all faculties. They received 5 euros for their participation, as the study lasted approximately 45 minutes. We followed APA ethical standards to conduct the present study.

### Natural language argument sets

We used natural language argument sets that we designed, constructed, and validated in two previously published studies [22, 28], so that the structure of these sets could be interpreted according to the intended *AFs*. Note that we selected the natural language arguments with attack types for which the agreement between the AFs we targeted and the evaluations from the two previous studies was the highest. The argument sets were designed to correspond to *AFSimple* (three arguments), *AFFloating* (four arguments) or *AF3-cycle* (five arguments). Natural language argument sets could be divided into three different thematic contexts: arguments based on news reports, arguments based on scientific publications, and arguments based on the precision of a calculation tool. In our further analyses, we decided not to analyze the context effect and to group them in their respective AF. As an example, here is the argument set of the scientific context corresponding to floating reinstatement:

*A*. *Specimen A consists only of amylase*. *The 1972 Encyclopedia of Biochemistry states that amylase is an enzyme*. *So specimen A consists of an enzyme*.
*B*. *A peer-reviewed research article by Smith et al*. *from 2006 presented new findings that amylase is not an enzyme*. *Therefore no specimen consisting only of amylase consists of an enzyme*.
*C*. *A study that the Biology Laboratory of Harvard University has published in 2011 corrects mistakes made in the study by Smith et al*. *and concludes that amylase is a biologically active enzyme*.
*D*. *A study that the Biochemistry Laboratory of Oxford University has published in 2011 corrects mistakes made in the study by Smith et al*. *and concludes that amylase is a biologically inactive enzyme*.

Each argument within a given set was labeled with a letter, starting from *A*. For every argument set {*A*, *B*, *C*, *D*} representing floating reinstatements, we used the subset {*A*, *B*, *C*} as an argument set representing simple reinstatements, so the data on simple and floating reinstatements can be directly compared. In total, there were three arguments sets for *AFSimple* and *AFFloating*, since there were three thematic contexts. However, due to the particular structure of *AF3-cycle*, and due to the difficulty of adequately designing an argument set of this complexity and verifying its correspondence to the *AF3-cycle* through pilot studies, we could only include a single argument set corresponding to the *AF3-cycle* in this study, which was of scientific context. The selection of natural language argument sets is available in S2 File.

### General procedure

Participants answered the questionnaire in groups of three to five students. Importantly, the members of a given group were presented with the same set of three to five natural language arguments, as a function of the number of members in the group (i.e., groups of 3 for *AFSimple*, groups of 4 for *AFFloating*, and groups of 5 for *AF3-cycle*). Five different groups evaluated each argument set. Overall, 45 participants responded to the three *AFSimple* sets (15 participants for each of the scientific, mathematical, and news report context sets), 60 participants responded to the three *AFFloating* sets (20 participants for each of the scientific, mathematical, and news report context sets), and 25 participants responded to the unique *AF3-cycle* set.

The study consisted of one questionnaire divided into two successive parts. After the instructions, we displayed a target set of natural language arguments that the participants had to carefully read before responding. The successive parts of the questionnaire were the following: In the first part, participants were instructed to draw the attack relations between the given arguments. After that, in the second part, they were told to judge the acceptance of each argument in light of the information provided in all arguments. Participants had to evaluate the same natural language arguments (and thus the same *AF*s) across both parts. Both parts similarly combined individual and collaborative aspects. These parts are described in more detail in the following sections.

#### Part A: Draw attack relations between arguments

In this first part, participants had to provide a graphic depiction of the attacking relation between each argument from the set. They were instructed to write down the letters of the arguments (*i*.*e*., *A*, *B*, *C*) within a blank box and then explicitly represent the relation between every argument pair: if they considered that one argument was attacking another one, they had to draw an arrow from the former pointing to the later. An arrow (or the absence thereof) from a given drawing was considered as correct if the corresponding *AF* (*AFSimple*, *AFFloating*, or *AF3-cycle*, see Fig 1) expected an attack (or the absence thereof). Within the instructions, participants were given illustrations of the three possible attacking relations between two natural language arguments: a *unidirectional attack* from one argument to another, a *bidirectional attack* between two arguments, and *no attack* between them (see S2 File).

Importantly, as mentioned in the introduction, our method mixed personal and collective phases to favor elaborated reasoning: in a first individual step, participants had to draw and then evaluate on a five-point Likert scale whether they subjectively thought their drawing was correct (without looking at the drawings of others). In a second collaborative step, after everyone had finished responding, all group members had to discuss their initial responses with each other in order to reach a consensus and provide one response as a group (or as a function of the simple majority if no consensus could be reached). In a third and final individual step, participants had the opportunity to follow–or not to follow–the group decision by giving their final answer, and they had to provide an ultimate evaluation of their subjective confidence in the accuracy of their latest response (also on a five-point Likert scale, and also without looking at the final responses of others). After responding to part A, they could start responding to the second part.

#### Part B: Evaluate the acceptability status of every argument

Participants were here asked to judge the acceptability status of each argument from the argument set that they had to draw in part A. They were explicitly instructed that they should not base their judgment on their knowledge, but only on the content of the arguments, and that, by default, an argument should be accepted, unless the other arguments provide reason to reject it. With the help of three examples (see S2 File), they were instructed to *accep*t non-conflicting arguments (*no attack*), to *reject* one argument when another one provides reason to reject it (*unidirectional attack*) and to consider both arguments *undecided* when there is a symmetric conflict between them (*bidirectional attack*). We only provided examples that consisted of a pair of arguments for which all standard semantics agree on the justification status of each argument (following Baroni & Giacomin [29, 30]), so that they did not prime the participants in the direction of any of these semantics. For every argument, participants were instructed to indicate, by ticking the relevant box, either that they *accept* the argument, that they *reject* it, or that they consider it *undecided*.

We also used here a method mixing personal and collective responses as in Part A; the acceptability status evaluation involved a three-phase process with a first individual judgment, followed by a group discussion, and then a final individual response. Similarly, at the end of every personal step, participants had to evaluate how they subjectively felt their response to be correct on a five-point Likert scale. We analyzed and reported the subjective confidence data in S3 File. Participants were unaware of each other’s responses during the personal stages. To discourage guessing, they were told that there was one “correct” response for each argument set, although the standard semantics provide different interpretations, and thus different expected responses, for *AFFloating* and *AF3-cycle*. There was thus no objectively correct response in this part, but we let our participants think there was one correct response. If the participants knew that there was no correct response for some of the *AF*s, they might have been less motivated in finding a solution, which would have drastically impaired their reasoning process [31].

It is noteworthy that partial data from Part B were previously presented in a conference paper [28].

## Results

### Part A. Attack drawings

**Fig 2:**
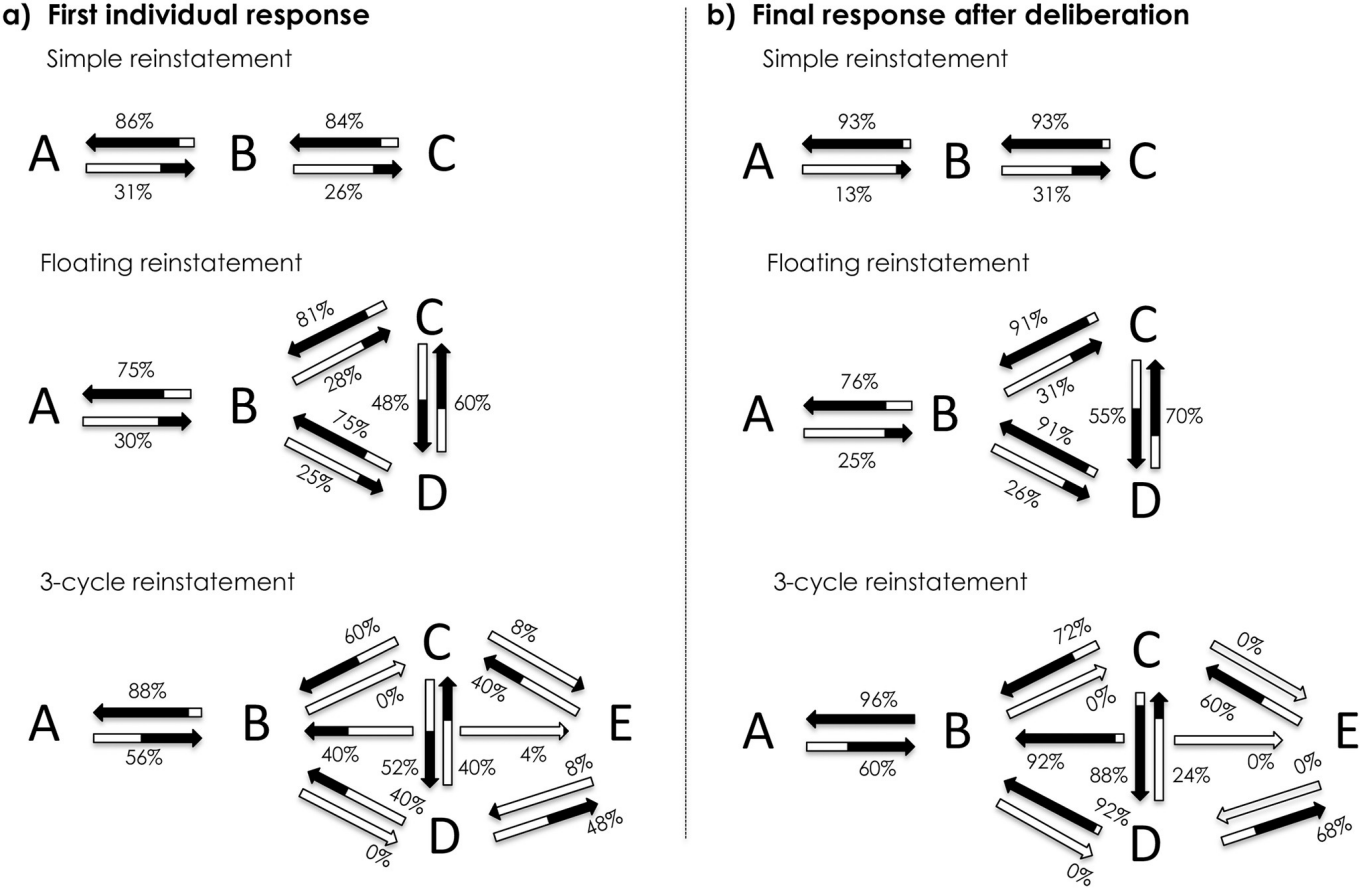
Graphical depiction of the relevant attacks between the arguments (displayed as a letter) from the three argumentation frameworks (*AFSimple*, *AFFloating*, *AF3-cycle*, following Fig 1). Each directional arrow depicts one attack from an argument to another, and is filled as a function of the percentage of participants who drew the corresponding attack in their response sheet during A) the first personal response phase, and B) the final individual step after the group deliberation phase.

In this part, participants drew attack arrows between the arguments. We computed the percentage of participants who drew a particular arrow from an argument to another for every attack relation within the argument sets. If the related theoretical *AF* predicted the existence of a specific attack relation, then drawing the corresponding arrow was the correct (*i*.*e*., expected) answer. Conversely, if no attack was theoretically foreseen, then no arrow was predicted. By computing these percentages, we highlighted the general tendencies across participants and extracted the majority choices. We then verified whether they consensually agreed with the predictions made by the three frameworks considered in this study (see Fig 1). We assessed whether the percentage of participants who drew a specific arrow was statistically larger than the chance level (*i*.*e*., larger than 50%, which was the absolute majority threshold) by conducting exact binomial tests. Note that the results are presented in terms of the accuracy of the arrows, not the accuracy of the entire graph. The general tendencies relating to the relevant attack relations are depicted in Fig 2, disregarding the thematic context. To avoid overloading the illustration, we did not graphically represent the irrelevant attack relations (*i*.*e*., relative to arguments that were theoretically independent of each other, such as the relation between argument *A* and argument *C*). However, all attack relations are considered in our further statistical analyses.

For simple reinstatement, participants correctly drew the arrows in 82.96% of the cases, 95% Confidence Interval (CI) [72.46, 93.46]. Their performance was even better after the group deliberation phase, reaching 89.25%, 95% CI [77.89, 100]. A unilateral paired t-test confirmed that this improvement was statically significant, *t*(5) = 2.13, Cohen’s *d* = 0.87, *p* = .042. Additionally, the simple majority of participants drew the expected attack relations. Exact binomial tests revealed that all majority choices (from both individual and collective phases) were significantly larger than the chance level (*i*.*e*., larger than 50%), with the highest *p* = .016. In other words, participants consensually agreed with the *AF* predicting attack relations for simple reinstatement.

For floating reinstatement, the accuracy improved from 77.36%, 95% CI [67.98, 86.74] to 82.50%, 95% CI [73.25, 91.74] after the group deliberation step, which was significant, *t*(11) = 2.86, *d* = 0.82, *p* = .007. The majority of participants drew the predicted attack relations, except for one situation: in their first personal drawing, participants mostly did not point out the attack from argument *C* to argument *D*. However, after collaborative thinking, the majority correctly responded to the predictions of the framework, although exact binomial tests revealed that the 55% choice in favor of the existence of an attack from *C* to *D* still did not reach the significance level, *p* = .519. Except for this situation, all majority choices for every other attack relations were significantly larger than 50%, all *p*s < .001. Participants thus mostly agreed with the theoretical *AFFloating*, even if there was no substantial consensus on the bilateral attack relation between the arguments C and D.

For 3-cycle reinstatement, participants were initially correct in 77.40% of the cases, 95% CI [66.71, 88.08]. Their performance increased after the discussion phase, reaching 88.12%, 95% CI [79.28, 96.96]. Once again, the improvement due to the collaborative thinking was significant, *t*(19) = 4.34, *d* = 0.97, *p* < .001. By looking at the last majority choices, it seems that participants overall conformed to the theoretical predictions from *AF3-cycle*, except that they considered that there exists an attack from argument *A* to argument *B*. However, binomial tests revealed that this unexpected tendency did not reach the significance level, *p* = .307. These tests also revealed that the majority choices relative to two attack relations were not significant, from E to C, *p* = .305, and from D to B, *p* = .833). Other majority choices were significantly above the 50% threshold, with the largest *p* = .022.

Finally, it is worth mentioning that all percentages of drawn arrows where the AF under consideration actually predicted an attack (highlighted in Fig 1) improved after group discussion. Participants initially detected an attack relation in 63.92% of the expected cases, 95% CI [52.57, 75.27], whereas after deliberation they correctly drew 80.15% of the attack relations, 95% CI [71.34, 88.96]. A unilateral paired t-test confirmed that this performance was better after group discussion, *t*(12) = 4.24, *d* = 1.17, *p* < .001. However, when the relevant *AF* did not predict an attack, participants were efficient in refraining from drawing anything; their first response was correct in 79.75% of the cases, 95% CI [68.59, 90.92], and their last drawing was correct in 81.42% of the cases, 95% CI [69.31, 93.43]. This small improvement was not significant here, *t*(11) = 0.69, *d* = 0.19, *p* = .748). In other words, participants were able to correctly dismiss an attack when there was no theoretical reason to consider its existence. However, participants were less efficient in identifying an attack relation during the first personal drawing phase, but they reached a satisfactory level of response after collective deliberation: they indeed judged the attack relations according to our model prediction in 80.76% of the cases, which is quite high and consistent with observations from our pilot studies.

### Part B. Acceptability status evaluations

#### Simple majority choices

**Fig 3:**
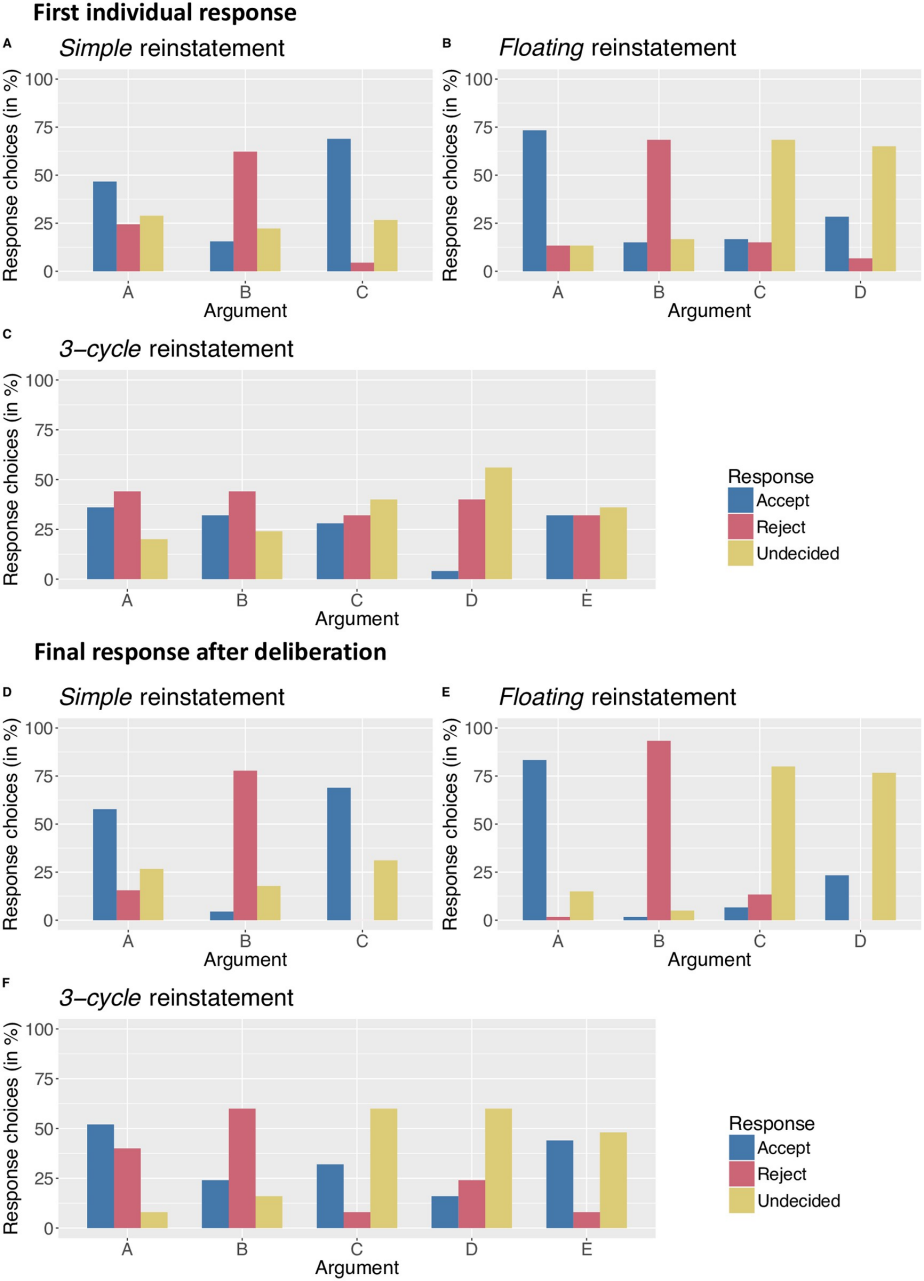
Percentage of responses in which participants chose to *accept* a corresponding natural language argument, to *reject* it, or to consider it *undecided*, for the three *AF*s. Data are provided for the first personal evaluation (upper part) and the last response after the group deliberation phase (bottom part).

In the introduction, we explained that each of the three semantics of Abstract Argumentation that we considered in this study (i.e., *grounded* semantics, *preferred* semantics, and *CF2* semantics) defines a justification status of *strongly accepted*, *strongly rejected*, or *undecided* for each argument from a given *AF*. This justification status could directly be compared to the responses provided by the participants in this task. Crucially, the three semantics provide distinct predictions about the acceptability of the arguments–except for the simple reinstatement on which they all agree–so that we could assess whether human evaluations were in line with one (or more) semantics. We thus computed the percentage of response choices among the three possibilities (i.e., *accept*, *reject*, or *undecided*) for each natural language argument within each AF (*AFSimple*, *AFFloating*, and *AF3-cycle*). Fig 3 depicts the data from the initial individual judgment and the final individual evaluation after group deliberation (mixing the three thematic contexts). From the observation of Fig 3, it appears that (simple) majority choices (*i*.*e*., choices larger than the other two possibilities) were consistent across the two response phases, with the only exception being the judgment of argument A within *AF3-cycle* (it was mostly rejected before the group deliberation, but in majority accepted afterward). As there were no “correct” responses in this part, we could not compute any “improvement” from the first personal choice to the decision after deliberation.

For simple reinstatement–for which all semantics predictively agree on the acceptability status–the simple majority of participants *accepted* arguments *A* and *C*, but *rejected* argument *B*, in both evaluations. The majority thus coincided with the predictions of all semantics. In order to statistically assess that the participants did not respond at random, and subsequently to check that a given majority choice was intended, we conducted Pearson chi-squared tests to verify whether the distributions of response choices differed from the uniform distribution. Only the choices for Argument A during the first phase did not significantly differ from the uniform distribution, *χ*^*2*^(2) = 3.73, *ϕ* = .24, *p* = .154. However, after deliberation, the distributions were not due to chance, minimal *χ*^*2*^(2) = 12.93, *ϕ* = .53, *p* < .001. This means that participants’ judgments were not random, and visual inspection of Fig 3 shows that participants substantially converged in evaluating the acceptance of the arguments from the simple reinstatement, and this convergence was in agreement with the three semantics.

In argument sets with floating reinstatement, participants’ ratings of the acceptability status of arguments were not random, as all distributions were significantly different from the uniform distribution, minimal *χ*^*2*^(2) = 31.30, *ϕ* = .72, *p* < .001, for both phases. Visual inspection of Fig 3 shows that the majority of participants tended to *accept* argument *A*, *reject* argument *B*, and consider arguments *C* and *D* as *undecided*, before and after group discussion. Contrarily to the situation for *AFSimple*, the three standard semantics provide different predictions here. The observed pattern of response choice from the majority of participants was theoretically expected by the *preferred* and *CF2* semantics, but did not match with the predictions from the *grounded* semantics.

Finally, for 3-cycle reinstatement, before collective thinking, the majority of participants *rejected* arguments *A* and *B* and considered the others as *undecided*. However, Pearson chi-squared tests revealed that only the distribution of the responses for argument *D* was significantly different from the uniform distribution, *χ*^*2*^(2) = 10.64, *ϕ* = .84, *p* = .004. All other response choices were at the chance level, maximal *χ*^*2*^(2) = 2.24, *ϕ* = .38, *p* = .326. The analyses thus revealed that there were no clear tendencies before the group deliberation phase. However, after discussion, the choices were not due to chance anymore, as the distributions of judgments were significantly different from the uniform distribution, minimal *χ*^*2*^(2) = 7.28, *ϕ* = .69, *p* = .026. Visual inspection of Fig 3 shows that the majority of participants agreed in *accepting* argument *A* and *rejecting* argument *B*, while considering every other argument as *undecided*. This is in accordance with the predictions from the *CF2* semantics.

#### Predicting data from semantics

In the previous section, we highlighted the general tendencies, indexed by the simple majority choices. In this section, we assess whether any of the three semantics could predict individual responses. In order to evaluate responses that every participant made in part B, we had to compare them to the predictions about acceptance and rejection of arguments that can be made based on Abstract Argumentation Theory. Given that participants had to draw an *AF* in part A, there were multiple possibilities about which *AF* to apply the semantics to. For example, one could compare the participant’s response in part B to the predictions that the various semantics make when applied to the *AF* that we intended, but one could also compare it to the predictions that the various semantics make when applied to the *AF* that participants produced in their last response in part A. We, therefore, considered three possibilities for the selection of the predictive argumentation framework to which we applied the three semantics when evaluating the responses from part B:

- The AF we intended for the argument set under consideration, as depicted in Fig 1. We refer to this AF as *BaseAF*.
- The AF that the participant under consideration gave as their last personal response in part A. We refer to this AF as *IndAF*.
- The AF that the group of the participant under consideration collectively provided in part A. We refer to this AF as *GroupAF*.

One could apply the three semantics to each of these three *AF*s. This gave rise to a total of nine predictors for the human acceptability evaluation in part B. It is important to note that two of the predictors (*IndAF* and *GroupAF*) depended on the response provided in part A, so that their predictions could thus differ from one participant to another. The percentage of correct predictions is reported in Table 2. Overall, the best predictor for all three responses was *BaseAF-CF2*. *BaseAF-CF2* correctly predicted the first personal response in 59.6% of all instances, the group response in 73.6%, and the final response in 72.4%. In other words, across all responses, the evaluation of arguments in part B was best predicted by applying *CF2* semantics to the AF that we intended. Note that there were three possible responses (*accept*, *reject* and *undecided*), so the chance level for a correct prediction was 33%. We conducted exact binomial tests that confirmed that all predictions from *BaseAF-CF2* were significantly above the chance level, all *p*s < .001.

**Table 2:** Percentage of correct predictions of the three semantics (*grounded*, *preferred*, and *CF2*) applied to the three AFs *(BaseAF*, *IndAF*, *GroupAF)* for the three responses provided in part B.

		*BaseAF*	*IndAF*	*GroupAF*
First response	*Grounded*	44.4%	48.6%	48.6%
	*Preferred*	57.8%	54.6%	55.2%
	*CF2*	59.6%	55.4%	56.4%
Group response	*Grounded*	49.4%	-	57.6%
	*Preferred*	68.6%	-	67.8%
	*CF2*	73.6%	-	69.8%
Final response	*Grounded*	49.2%	55.4%	48.6%
	*Preferred*	68.0%	63.4%	55.2%
	*CF2*	72.4%	63.6%	56.4%

Note. We thereafter refer to the different predictors by combining the naming system of the *AF* to the one of the semantics (*e*.*g*., *BaseAF-grounded* refers to the *grounded* semantics applied to the theoretical *AF* we intended). *IndAF* (referring to the personal response from every member of the same group in part A) could not predict the group response in part B, so there was no prediction in this case.

One noteworthy observation is that the *CF2* semantics yielded the best predictors for all three *AFs*. The *grounded* semantics systematically yielded by far the worst predictors of all three semantics. Another noteworthy observation is that in both *CF2* and *preferred* semantics, *BaseAF* yielded the best predictors out of all three AF*s* (the fact that it yielded a bad predictor in *grounded* semantics, on the other hand, seemed irrelevant given that the *grounded* semantics yielded the worst predictors anyway). The differences between the predictors might at first sight not give the impression of being substantial, but they were actually larger than it seemed. The reason for this is that in many cases, the predictors make the same prediction, so to evaluate the significance of the difference between any two predictors, we had to focus on those instances where the two predictors differed. Given that the set of arguments on which two predictors differed is a different set for any chosen pair of predictors, this way of comparing predictors did not provide a single scale on which all predictors can be compared, but instead, this comparison process had to be done separately for each pair of predictors. We described here below how *BaseAF-CF2* compared in this way to the second-best predictors, *BaseAF-preferred* on the one hand, and to *GroupAF-CF2* on the other hand.

As explained in the introduction, the only difference in the predictions between *CF2* and *preferred* semantics when applied to the three base AFs we intended was on the arguments *A* and *B* in *AF3-cycle*. Each of these two arguments was evaluated by five groups of five participants each (*i*.*e*., 25 human judgments for each argument). Of the first fifty individual responses in part B, twenty were in agreement with *BaseAF-CF2*, eleven were in agreement with *BaseAF-preferred*, and nineteen did not agree with either of them. Exact binomial tests revealed that the prediction rate was not significantly above the chance level, *p* = .182. However, of the fifty final personal responses, twenty-eight were in agreement with *BaseAF-CF2*, only six were in agreement with *BaseAF-preferred*, and sixteen did not agree with either of them. The prediction from *BaseAF-CF2* after group discussion was significantly above the chance level, while the one from *BaseAF-preferred* was significantly below, both *ps* < .001. In other words, *BaseAF-CF2* was significantly better than *BaseAF-preferred* in predicting human responses after collaborative deliberation.

Secondly, and similarly, the two predictors *BaseAF-CF2* and *GroupAF-CF2* had different predictions for 158 individual responses. Of the 158 first personal responses, seventy-five were in agreement with *BaseAF-CF2*, fifty-nine were in agreement with *GroupAF-CF2*, and twenty-four did not agree with either of them. After the collective phase, eighty-nine were in agreement with *BaseAF-CF2*, fifty-nine were in agreement with *GroupAF-CF2*, and ten did not agree with either of them. The predictions from *BaseAF-CF2* were significantly above the chance level for both individual and collaborative responses, both *p*s < .001. However, predictions from *GroupAF-CF2* were not significantly above this threshold, both *p*s = .141. Once again, *BaseAF-CF2* yielded the best predictors. We made similar comparisons between *BaseAF-CF2* and the remaining predictors to confirm that *BaseAF-CF2* yielded the best predictions in comparison to any of the other predictors in a statistically significant way, all *p*s < .001. Taken together, our data show that the *CF2* semantics applied to the theoretical AF we intended was substantially the best to predict the human acceptability status of the arguments.

## Discussion

In this study, we aimed at testing the cognitive plausibility of Formal Argumentation by conducting an empirical study on how young adults reason about conflicting information. Each questionnaire was based on a set of carefully designed natural language arguments. In the first part of the questionnaire, participants were instructed to draw the attack relations between the arguments from a given argument set. In the second part of the questionnaire, participants had to evaluate the acceptability status of each of the natural language arguments from the same set. Both parts involved individual and collective phases to favor rational thinking. We hypothesized that the responses provided by the participants would be–or become throughout the discussion–in line with our predictions based on Formal Argumentation. If this was the case, we intended to assess which of three standard semantics (*grounded*, *preferred*, and *CF2*) most adequately predicted how young adults judged the acceptability status of conflicting natural language arguments.

### Cognitive plausibility of Formal Argumentation

In this section, we verified whether the results from the main study support our first hypothesis that human behavior conforms to theoretical predictions from Formal Argumentation, more precisely to predictions based on *ASPIC+*. Drawing data reveal that participants consensually agreed with *AFSimple* and *AFFloating*, and that these agreements coincided with the theoretical predictions. For the more complex *AF3-cycle*, participants, the drawings were mostly in line with the predictions, except for the link between arguments *A* and *B*, for which the participants tended to judge the relation as a bilateral attack whereas we intended it as unilateral. As explained in the method section, due to the intrinsic complexity of the *AF3-cycle*, we were not able to design an argument set that strictly followed the same rules as the other sets. More precisely, the argument set that corresponded best to *AF3-cycle* in the pilot studies had the property that the attack from argument *B* to argument *A* was unidirectional only due to the recency of the publications cited in argument *B*. Already in our previous study [22], we found that giving preference to an argument based on recency of a cited publication was not as good a criterion for unidirectionality of an attack as the other criteria that we used. The limitation of these items was supported by the findings in part A of the current study, as participants tended to judge the attack to be bidirectional. However, this was the only situation where the results were not in line with the theoretical predictions. In more than 80% of the cases, the judgment of the existence and the directionality of the attack relations across all *AF*s was correct, consistent with previous observations [22, 28].

Our results thus clearly show that humans were efficient in drawing arrows corresponding to the theoretical attack relations between natural language arguments predicted by Formal Argumentation. There are two implications for this result. From a theoretical perspective, the data supports Formal Argumentation as a good model of human processing of conflicting natural language arguments, because it predicts very well which conflicts between arguments and which directionality of these conflicts young adults identify after group deliberation. Secondly, this is to the best of our knowledge the first evidence that adults from various backgrounds can graphically reproduce attack relations between natural language arguments. This suggests that adults not only understand the content and the relations between the arguments but are also able to depict an abstract representation of their content and relations. One can thus conclude that the participants were able to grasp the meaning of such graphical depictions. The use of such illustrations might be an interesting way to assess human interpretation of conflicting information without the need for natural language arguments. This is very promising for future studies on argumentative reasoning that could use such graphical tools in their method.

The second piece of evidence in favor of our hypothesis comes from the acceptability status evaluation for the argumentation framework *AFSimple* where the three standard semantics actually agree on the predictions. In other words, for this simple *AF* there was only one answer predicted by Formal Argumentation, and we could thus verify whether the acceptability status judgments conformed to this theoretical prediction. All semantics predict that arguments *A* and *C* should be accepted, whereas they foresee that argument *B* should be rejected; and this was exactly what we found for the simple majority choice, before and after group discussion. Humans thus evaluated the acceptability status of our natural language arguments as predicted by the three standard argumentation semantics. Given that reinstatement is one of the most fundamental features of Dung-style Abstract Argumentation, this finding speaks in favor of the cognitive plausibility of Dung-style theory. Of course, further argumentation frameworks on which all semantics agree will have to be considered in future studies in order to test whether this holds in general, or whether it only holds in limited cases.

### Predictive capacity of different semantics

We showed in the previous section that the responses provided by the participants conformed to our theoretical predictions based on Formal Argumentation, corroborating our first hypothesis. We now check which of the three standard semantics (*grounded*, *preferred*, and *CF2*) was the best predictor of the human thinking process during our task. Noticeably, as the three considered semantics do not provide any information about the existence of an attack relation within a given *AF*, we can only interpret our second hypothesis in the light of the results from part B. In other words, we verify here whether one semantics adequately predicted how human participants judged the acceptability status of conflicting natural language arguments. The majority choices were overall in line with *preferred* and *CF2* semantics. We conducted additional analyses aiming at directly comparing the predictive capacity of each semantics by focusing pairwise on the arguments on which two semantics have different justification status. The results showed that independently of the thematic context, *CF2* and *preferred* semantics were better predictors for human argument acceptance than the *grounded* semantics. Overall, our findings suggest that *CF2* semantics predicted human argument acceptance better than *preferred* semantics, but the data for this comparison were limited to a single thematic context. These findings suggest that the principle of conflict-free directional decision making encoded in the CF2 semantics may explain human judgments on the acceptability of arguments better than the principle of defending all accepted arguments that is encoded in the grounded and preferred semantics.

Note that our findings on simple reinstatement and floating reinstatement could be seen as a confirmation of the findings of Rahwan *et al*. [14] on human judgments related to these two argumentation frameworks. Even though the data were not directly comparable since their participants judged arguments on a 7-point Likert scale instead of making the three-valued acceptability judgments that our participants were asked to make, their final interpretation of their data was similar to ours: the predictions of standard semantics on *AFSimple*, were in line with human judgments, and in *AFFloating*, *preferred* semantics predicted human judgments better than *grounded* semantics. We extended these findings with data from *AF3-cycle* suggesting that *CF2* semantics was an even better predictor for human judgments than *preferred* semantics.

Finally, given that the comparison between the *preferred* and *CF2* semantics that we conducted on the acceptability status only concerned arguments *A* and *B* from *AF3-cycle*, one could object that the participants who did not draw the intended attack relation between these arguments during part A did not correctly understand the relationship between these arguments, in which case our analyses here were flawed. However, additional analyses revealed that the best predictors of the acceptability status were the ones from our intended theoretical *AF*, and not the ones from the *AF*s that we constructed from either the individual or the collective response provided in part A. This means that our results did not support that participants responded in part B on the basis of their previous response, which interestingly suggests that they disregarded, or did not automatically see, the implication of the drawing phase on the evaluations in part B. Future studies focused on much more complex *AF* structures are still needed to understand how participants evaluate such a large number of conflicting natural language arguments. Nevertheless, within the scope of the current study, the data do not provide sufficient evidence that participants made their acceptability status from their drawing for *AF3-cycle*, so the data from part A does not weaken our interpretation of the predictive capacity of the *preferred* and *CF2* semantics.

### Collaborative enhancement

We did not intend to analyse in details the effect of collaborative discussion on our results, but we need to mention that the group method successfully allowed participants to provide a more rational response in the tasks. There was a substantial improvement in the accuracy of the drawing for each of the corresponding *AF*s, although it is worth mentioning that performance was already good for *AFSimple* and *AFFloating*. In these cases, the collaboration discussion let to even higher performance, which supports the beneficial effect of the collaborative step. For the more complex *AF3-cycle*, the enhancement was very pronounced: participants had trouble in detecting most of the attack relations at first sight but were overall efficient after the collective deliberation. In other words, our data are in favor of an objective improvement of the response accuracy after collaboration. Such “group superiority” has often been described in the literature, with the involvement of many cognitive and social factors such as the communication and the (re)formulation of ideas, and the critical evaluation of the choices individually made [32–35]. Interestingly, this enhancement reflects in the subjective confidence evaluations provided by the participants across the questionnaire: participants systematically felt they were more confident in the accuracy of their final response (i.e., after the deliberation) than they were *prima facie* (see S3 File). Responses in part A were thus both objectively and subjectively better after the discussion with peers, which supports our claim that this method involved mental processes found within collaborative learning [36].

Despite this enhancement, we must acknowledge as a limitation of the current study that the collaborative aspect of our study involved group dynamics that we did not control. Our method attempted to minimize group pressure during the initial and final response stages by making them individual (i.e., without knowing what each others responded at the same stage), but we cannot rule out that other group factors influenced individual responses. One such factor was the conformity/independence to/from the majority decision (following Asch, [37]). Our participants were indeed instructed to reach a consensus (if possible) during the discussion phase, and it is possible that some participants conformed to the majority assessment of the group. For these individuals, therefore, it is likely that their final response did not reflect their personal thinking so much as the judgment of the majority group.

### Conclusion and perspectives

This study contributes to the still young and under-explored research field of cognitive aspects of Formal Argumentation. Our data support that the normative accounts of Formal Argumentation are cognitively plausible, at least in a collaborative context where humans are engaged in thinking and reasoning together to find the solution. This study further provides critical insights into humans’ understanding of the attack relation between arguments and acceptance of conflicting information, which are critical skills in argumentative reasoning [7]. Formal Argumentation does thus provide a valuable framework to study how humans deal with new conflicting information.

The limitations of the presented study highlight potential further research in this field: Future studies should be based on a larger variety of *AF*s than the three frameworks used in this study. Given that our study suggests that naive-based semantics like *CF2* are good predictors of human judgments, special attention should be given to *AF*s on which the various proposed naive-based semantics (*CF2*, *stage*, *stage2*) have different predictions, so as to find out which one of them predicts human judgments best. Furthermore, future studies should attempt to minimize the influence of world knowledge on argument judgments, so as to get a clearer idea of the influence of the logical form of arguments on human judgments. One possible way in which this could be achieved is by using arguments that are embedded in a fictional setting about which the participants can have no knowledge other than the one provided to them during the experiment or directly provide them graphically illustration of the attack relation without any language.

## Supporting information

S1 File Definitions of the argumentation semantics under consideration. (DOCX)

S2 File Argument sets. (DOCX)

S3 File Additional analyses. (DOCX)
