# Supplementary material for: Reasoning on conflicting information: An empirical study of Formal Argumentation
Source: PLoS One. 2022 Aug 19;17(8):e0273225. doi: 10.1371/journal.pone.0273225 (PMC9390901; doi:10.1371/journal.pone.0273225)
Supplement: S1 File — (DOCX) [file pone.0273225.s001.docx]

S1. Definitions of the argumentation semantics
under consideration

In the main text, we focus on three standard argumentation semantics from the abstract argumentation literature, namely on *grounded semantics*, *preferred semantics*, and *CF2 semantics^[[1]](#footnote-1)^.* More precisely, we focus on the justification statuses of arguments concerning these semantics. We define here in more detail these three semantics as well as the notion of *justification status*.

*Grounded* semantics and *preferred* semantics are both based on the idea that each accepted argument must be defended by the extension (i.e., by the set of all accepted arguments). We say that a set *S* of arguments *defends* an argument *A* if every argument that attacks *A* is attacked by some argument in the set *S*. For example, in *AFFloating*, the set {*C*} defends argument *A*, because the only argument that attacks *A* (namely argument *B*) is attacked by *C*. Additionally, the set {*C*} defends the argument *C*, because the only argument that attacks *C* (namely argument *D*) is attacked by *C*. We say that a set *S* of arguments is *admissible* if it is conflict-free and *S* defends every argument in *S*. For example, in *AFSimple*, the set {*C*} is admissible, because clearly {*C*} is conflict-free and because {*C*} defends *C*.

An additional idea that is present in both *grounded* semantics and *preferred* semantics is that any argument that is defended by accepted arguments should also be accepted. We say that a set *S* of arguments is *complete* if *S* is admissible and every argument defended by *S* is an element of *S*. For example, in *AFSimple*, the set {*C*} is not complete, because {*C*} defends *A*, but *A* is not an element of {*C*}. The set {*A,C*}, on the other hand, is complete, because the only arguments it defends are *A* and *C*, and they are both elements of {*A,C*}. Additionally, the notion of completeness ensures the previously mentioned property that an argument that is not attacked gets accepted. The reason is that an argument that is not attacked is defended by every set. For example, in *AFSimple*, the set {*B*} is not complete, because {*B*} defends *C* and *C* is not an element of the set {*B*}. The reason that {*B*} defends *C* is that *C* is not attacked by any argument, so the rules of formal logic tell us that the statement that every argument that attacks *C* is attacked by *B* comes out true (in formal logic, a universal statement, *i.e.* a statement with *every*, is considered true when there is no element to which it is applicable).

We now define the *grounded* semantics and the *preferred* semantics by defining under which conditions a set of arguments is an extension of an argumentation framework in the *grounded* and *preferred* semantics respectively:

- The *grounded extension* of an *AF* is the minimal complete set of arguments in the framework. In other words, it is a complete set of arguments that cannot be reduced to a smaller complete set of arguments by removing some arguments from it.
- *A preferred extension^[[2]](#footnote-2)^* of an *AF* is a maximal complete set of arguments in the framework. In other words, a preferred extension is a complete set of arguments that cannot be extended to a larger complete set of arguments by adding more arguments to it.

We now state the result of applying these methods to the three argumentation frameworks presented above. These results can be easily verified by applying the definitions that we have presented.

- The set {*A,C*} is both the *grounded* extension and the only *preferred* extension of the simple reinstatement framework *AFSimple* from Figure 1a.
- The *grounded* extension of the floating reinstatement framework *AFFloating* from Figure 1b is the empty set (in other words, no argument gets accepted according to the *grounded* semantics), while there are two *preferred* extensions, namely {*A,C*} and {*A,D*}.
- In the case of the 3-cycle reinstatement framework *AF3-cycle* from Figure 1c, the empty set is both the *grounded* extension and the only *preferred* extension.

The goal of our empirical study is to compare human judgments about the acceptability of arguments with predictions provided by the standard argumentation semantics. However, for a naïve participant, it would be too complicated to produce multiple extensions (i.e., multiple alternative sets of arguments that are acceptable together). Instead of comparing their judgments directly to the extensions provided by the argumentation semantics, we compared the judgments to the *justification status* of the arguments according to various semantics (see Wu and Caminada, 2010, as well as Baroni et al., 2018), since the justification status is always unique for each argument. In particular, we focus on the justification statuses *strong accept* and *strong reject*, which can be defined as follows: An argument *A* is *strongly accepted* in an argumentation framework *AF* with respect to a semantics σ if *A* is in every extension that semantics σ assigns to the argumentation framework *AF*. An argument *A* is *strongly rejected* in an argumentation framework *AF* with respect to a semantics σ if every extension that semantics σ assigns to the argumentation framework *AF* contains an argument that attacks *A*.

Let us illustrate these notions by considering how they apply to the three *AF*s from Figure 1 (in the main text).

- In the case of *AFSimple*, arguments *C* and *A* are strongly accepted with respect to *grounded* and *preferred* semantics, and argument *B* is strongly rejected with respect to both semantics.
- In the case of *AFFloating*, argument *B* is strongly rejected and argument *A* is strongly accepted with respect to preferred semantics. Arguments *C* and *D* are neither strongly accepted nor strongly rejected with respect to *preferred* semantics. With respect to *grounded* semantics, none of the four arguments are either strongly accepted or strongly rejected.
- In the case of *AF3-cycle*, none of the five arguments is strongly accepted or strongly rejected with respect to both semantics.

Now we still need to define the *CF2 semantics*, which was first introduced by Baroni, Giacomin, and Guida (2005). The idea behind *CF2* is that we partition the *AF* into parts that are called *strongly connected components* and recursively evaluate the framework component by component. In each component, we choose a maximal conflict-free set of arguments (i.e., a conflict-free set that cannot be extended to a larger conflict-free set). Once an argument has been chosen, we remove all arguments that it attacks. We will now make this more precise by defining what a strongly connected component is and how the *CF2* extensions of the three considered AFs can be determined by evaluating the framework’s component by component.

Let *AF* be an argumentation framework. A component of *AF* is called *strongly connected* if there is a path of attack from each argument in the component to each other argument in it. A Str*ongly Connected Component* (SCC) of *AF* is a maximal component of *AF* that is strongly connected, which means it cannot be extended to a larger component that is also strongly connected. For example, {*C*,*D*,*E*} is an SCC of *AF3-cycle*, because every argument in {*C*,*D*,*E*} can be reached from any other argument in {*C*,*D*,*E*} by moving along the attack arrows, and because {*C*,*D*,*E*} cannot be extended to a larger set still having this property. In {*B*,*C*,*D*,*E*}, on the other hand, there is no path from *B* to *C* along the attack arrows (one may only follow the arrows according to the direction in which they are pointing). The other two SCCs of *AF3-cycle* are {A} and {B}, each of these two arguments forms an SCC by itself.

We now describe how the component by component evaluation works in the case of *AF3-cycle*. Because the SCC {*C*,*D*,*E*} is not attacked by any argument outside it, we can start the evaluation at this SCC. We choose a maximal conflict-free subset of {*C*,*D*,*E*}. Given that *C* attacks *D*, *D* attacks *E* and *E* attacks *C*, the only choices here are the subsets that consist of one argument each, i.e. the sets {C}, {*D*} and {*E*}. Now, whichever choice we make, we need to delete any argument outside {*C*,*D*,*E*} that is attacked by an argument in the chosen set. In all three cases, this will lead to argument *B* being deleted, because each of *C*, *D* and *E* attacks *B*. Given that *B* is deleted, we no longer need to evaluate the SCC {*B*}, and we can go directly to the SCC {*A*}, as it does not get attacked by an argument outside it (given that the only attacker, namely *B*, has already been deleted). Again, we need to choose a maximal conflict-free subset of the SCC under consideration. But the only maximal conflict-free subset of {*A*} is {*A*} itself. Wrapping up, this means that no matter which of the three possible choices {C}, {*D*} and {*E*} we make at the beginning, argument *B* always gets rejected and argument *A* always gets accepted. In other words, the three CF2 extensions of *AF3-cycle* are {*A*,*C*}, {*A*,*D*} and {*A*,*E*}.

In the case of *AFFloating*, the evaluation proceeds similarly: The three SCCs are {*A*}, {*B*} and {*C*,*D*}, and we start the evaluation at {*C*,*D*}, because this SCC is not attacked by an outside argument. The two maximal conflict-free subsets of {*C*,*D*} are {*C*} and {*D*}, and no matter which of the two we choose, argument *B* gets deleted and argument *A* gets accepted. So the two *CF2* extensions of *AFFloating* are {*A*,*C*} and {*A*,*D*}.

Finally, in the case of *AFSimple*, the evaluation process is somewhat simpler: The three SCCs are {*A*}, {*B*} and {*C*}, and we start the evaluation at {*C*}, because this SCC is not attacked by an outside argument. The only maximal conflict-free subset of {*C*} is {*C*} itself, so we accept *C*, delete *B* and finally accept *A*. In other words, the only *CF2* extension of *AFSimple* is {*A*,*C*}.

This component-by-component evaluation of *AF*s can be used to determine the *CF2* extensions of any finite *AF*. Since our description of the procedure was not fully formal, we additionally provide a formal definition of *CF2* semantics that follows the notation of Dvořák and Gaggl (2016) and that can be applied to both finite and infinite argumentation frameworks.

Given an Argumentation Framework *AF*, we denote the set of SCCs of *AF* by *SCCs*(*AF*). When two arguments *A* and *B* are in the same SCC, we write$\text{A}\sim\text{B}$. Given a subset *S* of the set of arguments of *AF*, the set of arguments$D_{AF}\left( S \right)$is defined as follows:

$$D_{AF}\left( S \right)\text{ }:=\text{ }\{B\text{ | there is an }A\text{ in }S\text{ such that }A\text{ attacks }B\text{ and not }A\sim B\}.$$

Now *CF2* extensions are recursively defined as follows:

Let *AF* be an argumentation framework and let *S* be a subset of the set of arguments of *AF*. Then *S* is a *CF2* extension of *AF* if either:

- *AF* has only one SCC, and *S* is a maximal conflict-free set of arguments, or
- *AF* has more than one SCC, and for each *C* in *SCCs*(*F*),$\text{S }\cap\text{ C}$ is a *CF2* extension of $AF\text{|}_{\text{C}-\text{D}_{\text{AF}}\left( \text{S} \right)}$

Given that there can be multiple *CF2* extensions, we apply the justification statuses defined to come to a single conclusion about each argument from our three example frameworks:

- In the case of *AFSimple*, arguments *C* and *A* are strongly accepted and argument *B* is strongly rejected with respect to *CF2* semantics (just as for *grounded* and *preferred* semantics).
- In the case of *AFFloating*, argument *B* is strongly rejected and argument *A* is strongly accepted with respect to *CF2* semantics, while arguments *C* and *D* are neither strongly accepted nor strongly rejected with respect to *CF2* semantics (just as for *preferred* semantics).
- In the case of *AF3-cycle*, argument *B* is strongly rejected and argument *A* is strongly accepted with respect to *CF2* semantics, while arguments *C*, *D* and *E* are neither strongly accepted nor strongly rejected with respect to *CF2* semantics. Here *CF2* semantics differs from both *grounded* and *preferred* semantics.

Table 1 (in the main text) summarizes the extensions and the justification status of the three semantics for each considered *AF*.

**Supplemental references**

Baroni, P., Gabbay, D., Giacomin, M., & van der Torre, L. (2018). *Handbook of Formal Argumentation*. College Publications.

Baroni, P., Giacomin, M., & Guida, G. (2005). SCC-recursiveness: a general schema for argumentation semantics. *Artificial Intelligence,* 168(1), 162–210.

Cramer, M., & Guillaume, M. (2019). Empirical Study on Human Evaluation of Complex Argumentation Frameworks. In *European Conference on Logics in Artificial Intelligence* (pp. 102-115). Springer, Cham.

Dvořák, W., Gaggl, S. A. (2016). Stage semantics and the SCC-recursive schema for argumentation semantics. *Journal of Logic and Computation,* 26(4), 1149–1202.

van der Torre, L., & Vesic. S. (2018). The principle-based approach to abstract argumentation semantics. In *Handbook of Formal Argumentation* (Eds. P. Baroni, D. Gabbay, M. Giacomin, & L. van der Torre). College Publications.

Wu, Y., Caminada, M. (2010). A Labelling-Based Justification Status of Arguments. *Studies in Logic*, 3(4), 12–29.

1. Further semantics have been considered in the Formal Argumentation literature (see Baroni, Gabbay, Giacomin, & van der Torre, 2018, for an overview). We briefly explain in this footnote why we have focused on these three semantics.

   Two additional semantics widely considered in the literature are the *complete* semantics and the *stable* semantics. The justification status in the *complete* semantics is the same as in the *grounded* semantics, so we do not consider *complete* semantics separately. The *stable* semantics has the serious disadvantage that for some *AF*s, there is no *stable* extension, so the justification statuses that we defined in the current study cannot be meaningfully applied to such frameworks.

   *CF2* semantics belongs to a family of semantics called *naive-based* semantics, to which one also counts *naive semantics*, *stage semantics* and *stage2 semantics* (see van der Torre and Vesic, 2018). Naive semantics ignores the directionality of attacks, a central feature that distinguishes argumentation theory from classical logic. *Stage* and *stage2* semantics, on the other hand, are much better-behaved and should in principle be considered as potential predictors of human argumentative reasoning. The only reason why we leave them out of the comparisons with the empirical data is that for the argumentation frameworks used in this study, they make the same predictions as *CF2* semantics, so the data from our experiment cannot distinguish between these three naive-based semantics. The reason why we chose to present CF2 rather than stage or stage2 in this paper is that another study that we conducted suggests that CF2 predicts human evaluation of arguments better than stage and stage2 (Cramer & Guillaume, 2019). [↑](#footnote-ref-1)
2. We wrote "*the* grounded extension" but "*a* preferred extension", as there is always a unique grounded extension, but there can be multiple preferred extensions. [↑](#footnote-ref-2)
