# Supplementary material for: Reasoning on conflicting information: An empirical study of Formal Argumentation
Source: PLoS One. 2022 Aug 19;17(8):e0273225. doi: 10.1371/journal.pone.0273225 (PMC9390901; doi:10.1371/journal.pone.0273225)
Supplement: S2 File — (DOCX) [file pone.0273225.s002.docx]

**S2. Argument sets**

## Part A Instructions

When an argument A can be used as a **counterargument** against another argument B, then we say that A **attacks** B. One can graphically depict the information that A attacks B as follows:

Another possibility for how two given arguments A and B can relate is that they can **attack each other**. This means that each one of them could be used as a counterargument against the other. Graphically it is depicted as follows:

When A and B are two arguments that **do not attack** each other, this is depicted by not having any arrow between A and B:

## Part A Example

A: Tweety is a bird, so Tweety can escape by flying over the fence.
B: Tweety is a penguin, so Tweety cannot fly.
C: Mary showed me a video of Tweety flying around in John’s bedroom, so Tweety can fly.

The attacks between the three arguments can be depicted as follows:

Arguments B and C **attack each other**, because each of them could be used as a counterargument against the other.

Argument B **attacks** argument A because B could be used as a counterargument against A. But A could not be used as a counterargument against B, so A **does not attack** B.

Between arguments A and C there is **no conflict** whatsoever, so none of them attacks the other.

## Part B Instructions

You need to evaluate each argument in relation to the other arguments to determine whether you accept or reject each argument.

Please tick one box per argument (i.e., per line). You need to make a choice for each argument. If the content of the arguments does not provide any reasons to accept or reject a given argument, tick the ”Undecided” box. It is important that you make your decision based on the arguments’ contents only, and not on your knowledge nor on any aspect that is not considered within the arguments. Accepting an argument means accepting all parts of the argument, not just the conclusion.

In general, an argument should be accepted, unless the other arguments provide reasons to reject it.

## Part B Examples

## *Example 1*

A. Specimen X is blue.

B. Specimen Y is green.

|  | Accept | Undecided | Reject |
| --- | --- | --- | --- |
| Argument A. | 🗹 | ☐ | ☐ |
| Argument B. | 🗹 | ☐ | ☐ |

There is **no attack** between the two arguments, so there is **no conflict** between the information provided by the two arguments. In other words, there is no reason to reject any argument. So one should **accept** both arguments.

## *Example 2*

A. Specimen X is blue. So specimen X is not yellow.

B. Specimen X is red. So specimen X is not yellow.

|  | Accept | Undecided | Reject |
| --- | --- | --- | --- |
| Argument A. | ☐ | 🗹 | ☐ |
| Argument B. | ☐ | 🗹 | ☐ |

Here the two arguments **attack each other**, as Specimen X cannot be blue and red at the same time. So even though the conclusion is the same, **we cannot accept** both arguments. There is no reason to prefer one argument over the other, so we are undecided which argument is correct and which one is wrong. So we put “**Undecided**” for both arguments.

## *Example 3*

Andrea: Elizabeth told me that Specimen X is yellow. So Specimen X is yellow.

Bernie: Elizabeth is a well-known liar. Andrea should not trust her.

|  | | Accept | Undecided | Reject |
| --- | --- | --- | --- | --- |
| Andrea. | ☐ | | ☐ | 🗹 |
| Bernie. | 🗹 | | ☐ | ☐ |

Here Bernie's argument attacks Andrea's argument, but Andrea's argument does not attack Bernie's argument, because Bernie provides a reason to reject Andrea’s argument, but Andrea does not provide a reason to reject Bernie’s argument. Therefore we **accept** Bernie’s argument and **reject** Andrea’s argument.

## Experimental sets

## Simple reinstatements

### Scientific context

**A**. Specimen A consists only of amylase. The 1972 Encyclopaedia of Biochemistry states that amylase is an enzyme. So specimen A consists of an enzyme.

**B**. A peer-reviewed research article by Smith et al. from 2006 presented new findings that amylase is not an enzyme. Therefore no specimen consisting only of amylase consists of an enzyme.

**C**. A study that the Biology Laboratory of Harvard University has published in 2011 corrects mistakes made in the study by Smith et al. and concludes that amylase is a biologically active enzyme.

### Mathematical context

**A**. We are given the information that x² = xy, and that x = 20+π−e^π^ . By dividing both sides of the first equation by x, we get that x = y.

**B**. By calculating 20+π−e^π^ on my calculator, I get that 20+π−e^π^ = 0. So one cannot divide an equation by 20+π−e^π^, because division by 0 is not allowed.

**C**. The computer program *Mathematica*, which does not make rounding errors like a simple calculator, computes that 20+π−e^π^ > 0.0009, so 20+π- e^π^ is a positive number, i.e. 20+π−e^π^ ≠ 0.

#### News report context

**A.** According to BBC, President Donald Trump shot an Asiatic lion yesterday. Asiatic lions generally have a mane. So President Donald Trump shot an animal that has a mane.

**B.** The website of the American Society of Animal Species explains that female Asiatic lions generally don't have a mane. So it is not right to say that Asiatic lion generally have a mane.

**C.** According to an article in America Today, the American Society of Animal Species is a pseudoscientific organization that is funded by a fundamentalist church and hires its staff based on church membership rather than scientific expertise. Therefore the explanations on its website cannot be trusted.

## Floating reinstatements

### Scientific context

**A**. Specimen A consists only of amylase. The 1972 Encyclopaedia of Biochemistry states that amylase is an enzyme. So specimen A consists of an enzyme.

**B**. A peer-reviewed research article by Smith et al. from 2006 presented new findings that amylase is not an enzyme. Therefore no specimen consisting only of amylase consists of an enzyme.

**C**. A study that the Biology Laboratory of Harvard University has published in 2011 corrects mistakes made in the study by Smith et al. and concludes that amylase is a biologically active enzyme.

**D**. A study that the Biochemistry Laboratory of Oxford University has published in 2011 corrects mistakes made in the study by Smith et al. and concludes that amylase is a biologically inactive enzyme.

#### Mathematical context

**A**. We are given the information that x² = xy, and that x = 20+π−e^π^ . By dividing both sides of the first equation by x, we get that x = y.

**B**. By calculating 20+π−e^π^ on my calculator, I get that 20+π−e^π^ = 0. So one cannot divide an equation by 20+π−e^π^, because division by 0 is not allowed.

**C**. The computer program *Mathematica*, which does not make rounding errors like a simple calculator, computes that 20+π−e^π^ > 0.0009, so 20+π- e^π^ is a positive number, i.e. 20+π−e^π^ ≠ 0.

**D**. The computer program *Maple*, which does not make rounding errors like a simple calculator, computes that 20+π−e^π^ < -0.0009, so 20+π−e^π^ is a negative number, i.e. 20+π−e^π^ ≠ 0.

#### News report context

**A.** According to BBC, President Donald Trump shot an Asiatic lion yesterday. Asiatic lions generally have a mane. So President Donald Trump shot an animal that has a mane.

**B.** The website of the American Society of Animal Species explains that female Asiatic lions generally don't have a mane. So it is not right to say that Asiatic lion generally have a mane.

**C.** According to an article in America Today, the American Society of Animal Species is a pseudoscientific organization that is funded by a fundamentalist church and hires its staff based on church membership rather than scientific expertise. Therefore the explanations on its website cannot be trusted.

**D.** According to an article in the Guardian, the American Society of Animal Species does not exist, and the website operating under this name is a hoax website run by a comedian. Therefore the explanations on this website cannot be trusted.

## 3-cycle reinstatement

**A.** Specimen A consists only of amylase. The 1962 Encyclopedia of Chemistry states that amylase is not an enzyme. So specimen A does not contain any enzymes.

**B.** The 2003 Encyclopedia of Biochemistry states that amylase is a glycosylase. The International Institute for the Advancement of Biochemistry has published new research results in 2006 that show that all glycosylases are hydrolases. The European Biochemistry Centre has published new research results in 2008 that show that all hydrolases are enzymes. Therefore any specimen consisting of amylase contains an enzyme.

**C.** The 2003 Encyclopedia of Biochemistry states that the institution running under the name "International Institute for the Advancement of Biochemistry" is not a serious scientific institution, so its publications cannot be trusted.

**D.** The International Institute for the Advancement of Biochemistry states that the institution running under the name "European Biochemistry Centre" is not a serious scientific institution, so its publications cannot be trusted.

**E.** The European Biochemistry Centre states that the 2003 Encyclopedia of Biochemistry contains many erroneous assertions and cannot be trusted.
