# Supplementary material for: Reasoning on conflicting information: An empirical study of Formal Argumentation
Source: PLoS One. 2022 Aug 19;17(8):e0273225. doi: 10.1371/journal.pone.0273225 (PMC9390901; doi:10.1371/journal.pone.0273225)
Supplement: S3 File — (DOCX) [file pone.0273225.s003.docx]

**S3. Additional analyses**

**Subjective confidence**

In both parts of the questionnaire, participants were instructed to subjectively rate after responding how they felt their responses were “correct” on a scale from zero to five.  In part A, the mean confidence evaluation of the first drawing was 2.33, 95% CI [2.29, 2.37].  Mean confidence judgment after the group deliberation phase increased and reached 3.22, 95% CI [3.18, 3.29].  In part B, the mean confidence judgment of the evaluations was initially at 2.32, 95% CI [2.24, 2.39].  After deliberation, the confidence level reached 3.10, 95% CI [3.03, 3.18].  Two unilateral paired sample *t*-tests confirmed that the confidence levels were significantly larger after the group discussion in both tasks, respectively *t*(129) = 10.60, *d* = 0.93, *p* < .001 and *t*(129) = 9.08, *d* = 0.79, *p* < .001.  This result indicates that participants subjectively felt that their response improved after group deliberation, supporting the beneficial effect of the collective phase on their rational thinking.
